# Supplementary material for: Practices and Awareness Regarding an Infant’s Sleep Environment among Japanese Caregivers: A Cross-Sectional Survey
Source: Int J Environ Res Public Health. 2024 Apr 12;21(4):471. doi: 10.3390/ijerph21040471 (PMC11050700; doi:10.3390/ijerph21040471)
Supplement: Supplementary file 1 [file ijerph-21-00471-s001.zip › ijerph-2917583-supplementary.pdf]

Supplementary Table S1 Survey Form. Questionnaire about Sleep Environment for Infants

<table>
<tr><td rowspan="3">The basic characteristics of the participant and their infant</td><td>1. How old are you (years)?</td><td>○ 10–19
○ 20–29
○ 30–39
○ 40–49
○ 50–59
○ 60–69
○ 70 or older</td></tr>
<tr><td>2. What relationship do you have with the infant?</td><td>○ Mother
○ Father
○ Grandmother
○ Grandfather
○ Other: ( )</td></tr>
<tr><td>3. How old is the infant (months)?</td><td>○ 0
○ 1
○ 2
○ 3
○ 4
○ 5
○ 6
○ 7
○ 8
○ 9
○ 10
○ 11</td></tr>
<tr><td rowspan="3">The current status of environment</td><td>4. Do you and the infant sleep in the same room?</td><td>○ 1. We always sleep in the same room.
○ 2. We usually sleep in the same room.
○ 3. We occasionally sleep in the same room.
○ 4. We sleep separately.</td></tr>
<tr><td>5. If you selected 1, 2 or 3 in Question 4, why do you sleep with the infant? (Please select all applicable answers or describe your reasons freely.)</td><td>□ I can become aware of the condition of the infant.
□ It is easy to take care of the infant.
□ I want to be close to the infant.
□ I do not have enough room to sleep separately.
□ Other: ( )</td></tr>
<tr><td>6. If you selected 4 in Question 4, why do you sleep separately? (Please select all</td><td>□ Other family member sleeps with the infant in the same room.
□ I want to encourage the infant's independence.</td></tr>
</table>

	applicable answers or describe your reasons freely.)	□ I have no special reason.<br>□ Other: ( )
	7. What kind of bedding do you use for the infant?	○ 1. Crib<br>○ 2. Futon for babies<br>○ 3. Bed for adults<br>○ 4. Futon for adults<br>○ 5. Other: ( )
	8. If you selected 3, 4 or 5 in Question 7, why did you select the answer? (Please select all applicable answers or describe your reasons freely.)	□ I thought a bed for adults would be good.<br>□ Although I wanted to use a crib, we had no space for the crib.<br>□ Although I wanted to use a crib, a crib was expensive.<br>□ Although I initially used a crib, I stopped using the crib.<br>□ Other: ( )
	9. If you selected 3, 4 or 5 in Question 7, does the infant sleep with you or other family member?	○ The infant always sleeps with me or other family member.<br>○ The infant usually sleeps with me or other family member.<br>○ The infant occasionally sleeps with me or other family member.<br>○ The infant sleeps alone.<br>○ I share a bed with the infant only when I put the infant to sleep.
	10. If you selected 3, 4 or 5 in Question 7, do you or other family member share (or plan to share) the same bedclothing to cover the infant?	○ Yes<br>○ No<br>○ Other: ( )
	11. If you selected 1 or 2 in Question 7, why did you select the answer? (Please select all applicable answers or describe your reasons freely.)	□ For comfort.<br>□ For safety.<br>□ For protection from a pet.<br>□ For individual use among siblings of similar ages.<br>□ The bedding was free (secondhand, etc.).<br>□ Other: ( )
	12. What do you use (or plan to use) as bedclothing to cover the infant in the cold season?	□ Heavy quilt that covers the infant.<br>□ Lightweight quilt that covers the infant.<br>□ Blanket.

	(Please select all applicable answers or describe your answer freely.)	□ None.<br>□ Other: ( )
	13. What do you use (plan to use) as the bedding surface for the infant in the cold season? (Please select all applicable answers or describe your answer freely.)	□ Firm futon/mattress.<br>□ Soft futon/mattress.<br>□ Blanket.<br>□ Other: ( )
	14. Is there something on the futon/bed on which the infant sleeps?	○ Yes<br>○ No
	15. If you answered "Yes" in Question 14, what is close to the infant in the shaded area in the figure? (Please select all applicable answers or describe your answer freely.)<br>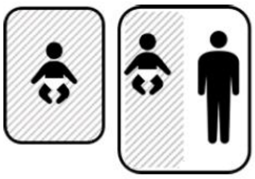	□ A pillow for the infant.<br>□ Items to take care of the infant (towels, cloths, diapers, etc.).<br>□ Stuffed toys/other toys.<br>□ Items for use by individuals other than the infant (cushions, smartphone, etc.).<br>□ Other: ( )
The participant's level of awareness	16. Did you seek sources of information to prepare the sleep environment for the infant before birth (i.e., during pregnancy)?	○ Yes<br>○ No<br>○ Did the same caregiving practices as for an older child
	17. If you answered "Yes" in Question 16, What sources of information did you seek to prepare the sleep environment for the infant before birth (i.e., during pregnancy)? (Please select all applicable answers or describe your answer freely.)	□ 1. Advice and experience of familiar people.<br>□ 2. Information obtained from the obstetrics and gynecology hospital (physicians, obstetric nurses, prenatal classes, etc.).<br>□ 3. Information obtained from local or national governments (maternal handbook, leaflets, prenatal classes, etc.).<br>□ 4. Information obtained from sources other than 1–3 (media, SNS, book/magazine about child rearing, personal website, etc.).<br>□ Other: ( )

	18. Among your answers for Question 17, select one source to which you referred the most.	○ 1. Advice and experience of familiar people.<br>○ 2. Information obtained from the obstetrics and gynecology hospital (physicians, obstetric nurses, prenatal classes, etc.).<br>○ 3. Information obtained from local or national governments (maternal handbook, leaflets, prenatal classes, etc.).<br>○ 4. Information obtained from sources other than 1–3 (media, SNS, book/magazine about child rearing, personal website, etc.).<br>○ Other: ( )
	19. Did you seek sources of information to prepare the sleep environment for the infant after birth?	○ Yes<br>○ No<br>○ Did the same caregiving practices as for an older child
	20. If you answered "Yes" in Question 19, What sources of information did you seek to prepare the sleep environment for the infant after birth (i.e., during pregnancy)? (Please select all applicable answers or describe your answer freely.)	□ 1. Advice and experience of familiar people.<br>□ 2. Information obtained from the obstetrics and gynecology/pediatrics hospital (physicians, obstetric nurses, prenatal classes, etc.).<br>□ 3. Information obtained from child care facilities (nurses, prenatal classes, etc.).<br>□ 4. Information obtained from local or national governments (maternal handbook, the program to visit all families with infants, prenatal classes, etc.).<br>□ 5. Information obtained from sources other than 1–3 (media, SNS, book/magazine about child rearing, personal website, etc.).<br>□ Other: ( )
	21. Among your answers for Question 20, select one source to which you referred the most.	○ 1. Advice and experience of familiar people.<br>○ 2. Information obtained from the obstetrics and gynecology/pediatrics hospital (physicians, obstetric nurses, prenatal classes, etc.).<br>○ 3. Information obtained from child care facilities (nurses, prenatal classes, etc.).<br>○ 4. Information obtained from local or national governments (maternal handbook, the program to visit all families with infants, prenatal classes, etc.).<br>○ 5. Information obtained from sources other than

		1–3 (media, SNS, book/magazine about child rearing, personal website, etc.).<br>○ Other: ( )
	22. How much do you care for the safety of the infant in sleep environment?	○ 5 ("Strongly aware")<br>○ 4 ("Aware")<br>○ 3 ("Neither aware nor unaware")<br>○ 2 ("Less aware")<br>○ 1 ("Completely unaware")
	23. A study introduced the ideal sleep environment for babies, as shown in the photo below [27]. Have you seen and/or heard of similar information as this image?<br>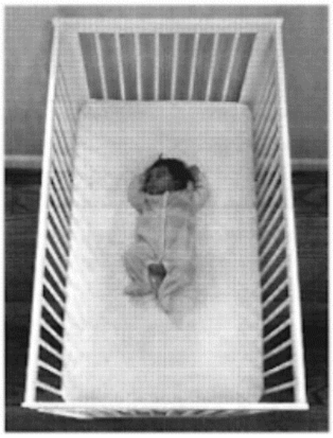	○ Yes<br>○ No
	24. Which organization do you think is the most reliable or should be reliable to learn about a safe sleep environment for infants?	○ Obstetrics and gynecology/pediatrics hospital.<br>○ Local or national governments.<br>○ Child care facility.<br>○ None.<br>○ Other: ( )
	25. Which measure do you think is most desirable to learn about a safe sleep environment for infants?	○ Distributing leaflets or flyers (Internet, books about child rearing, leaflets, etc.).<br>○ Watching instructional video (TV, YouTube, etc.).<br>○Face-to-face verbal instruction (support facility for child rearing, gynecology/pediatrics hospital, etc.).<br>○Joining hands-on workshops (prenatal classes, group activities for child rearing, etc.).<br>○ Other: ( )
